# Real-time and long-term monitoring of waves and suspended sediment concentrations over an intertidal algal reef

**DOI:** 10.1007/s10661-022-10491-0

**Published:** 2022-09-28

**Authors:** Trung Nguyen Ly, Zhi-Cheng Huang

**Affiliations:** Graduate Institute of Hydrological and Oceanic Sciences, National Central University, Taoyuan City, Taiwan

**Keywords:** Intertidal reef ecosystem, Long-term monitoring, Real-time monitoring, Suspended sediment concentration

## Abstract

Suspended sediment concentration (*c*) has been considered a critical environmental factor in reef habitats; however, the values and variations of *c* are not evident in a unique reef mainly created by crustose coralline algal concretions compared to abundant studies in coral reefs. The results of real-time and long-term monitoring of waves and *c* over the intertidal algal reef are reported because of the construction of an offshore industrial harbor near the reef. The real-time monitoring systems were based on techniques, including optical backscatter sensors (OBSs) for measuring *c*, pressure sensors for measuring waves, data loggers, and wireless networks for data transmission. The instruments sampled every hour and ran continuously and automatically for years. The OBS measurement was compared and validated with biweekly water sampling. A good correlation between the results of the two methods was observed. Nevertheless, more calibrations of OBSs in different seasons reduced the variance between the two methods over a year-long timescale. The year-long data showed a remarkable seasonal variation in *c*. The average *c* was approximately 140 mg/l during the winter season, while it was only approximately 70 mg/l during the summer season. The observed *c* was higher than that in other coral reef environments; the elevated and highly variable *c*, ranging from approximately 0 to 500 mg/l, may be one factor that creates the unique algae reef environment. The year-long measurement of waves and *c* showed that the variation in *c* was mainly due to the variation in waves in different seasons and was well correlated with the wave-induced bed shear stress. The real-time and long-term data measured by the system will aid in better understanding and providing useful environmental data for accessing future environmental changes and protecting reef habitats.

## Introduction

Suspended sediment concentration (*c*) and hydrodynamic conditions are essential factors in shallow coastal areas (Huang et al., 2012; Lu et al., 2017; Pang et al., 2020), especially in the presence of ecological systems, such as reefs (Jones et al., 2020). The roughness features of reef systems affect hydrodynamic processes and sediment properties (Pomeroy et al., 2017), which control *c* under various reef bottom conditions and help to prevent bed erosion (Fattahi Nafchi et al., 2022). Many studies have shown that waves and currents are two primary mechanisms driving sediment suspension and transport (Hsu et al., 2019; Pang et al., 2020; Voulgaris & Meyers, 2004). Waves start to shoal and interact with the seabed in shallow water, and waves might become a dominant driver of suspended sediment (Pomeroy et al., 2017, 2018; Voulgaris & Meyers, 2004; Wang et al., 2019). Another study showed that *c* was a function of bed shear stress (Nafchi et al., 2021). Some findings support that the total transport of sediment in coastal zones mostly depends on the wave mode (Buschmann et al., 2012; Pomeroy et al., 2018). Sediment suspension and hydrodynamics should both be considered together from the perspective of ecosystem recovery and restoration in coastal areas (Duarte et al., 2015; Fatahi Nafchi et al., 2021; Yu et al., 2020).

**Fig. 1:**
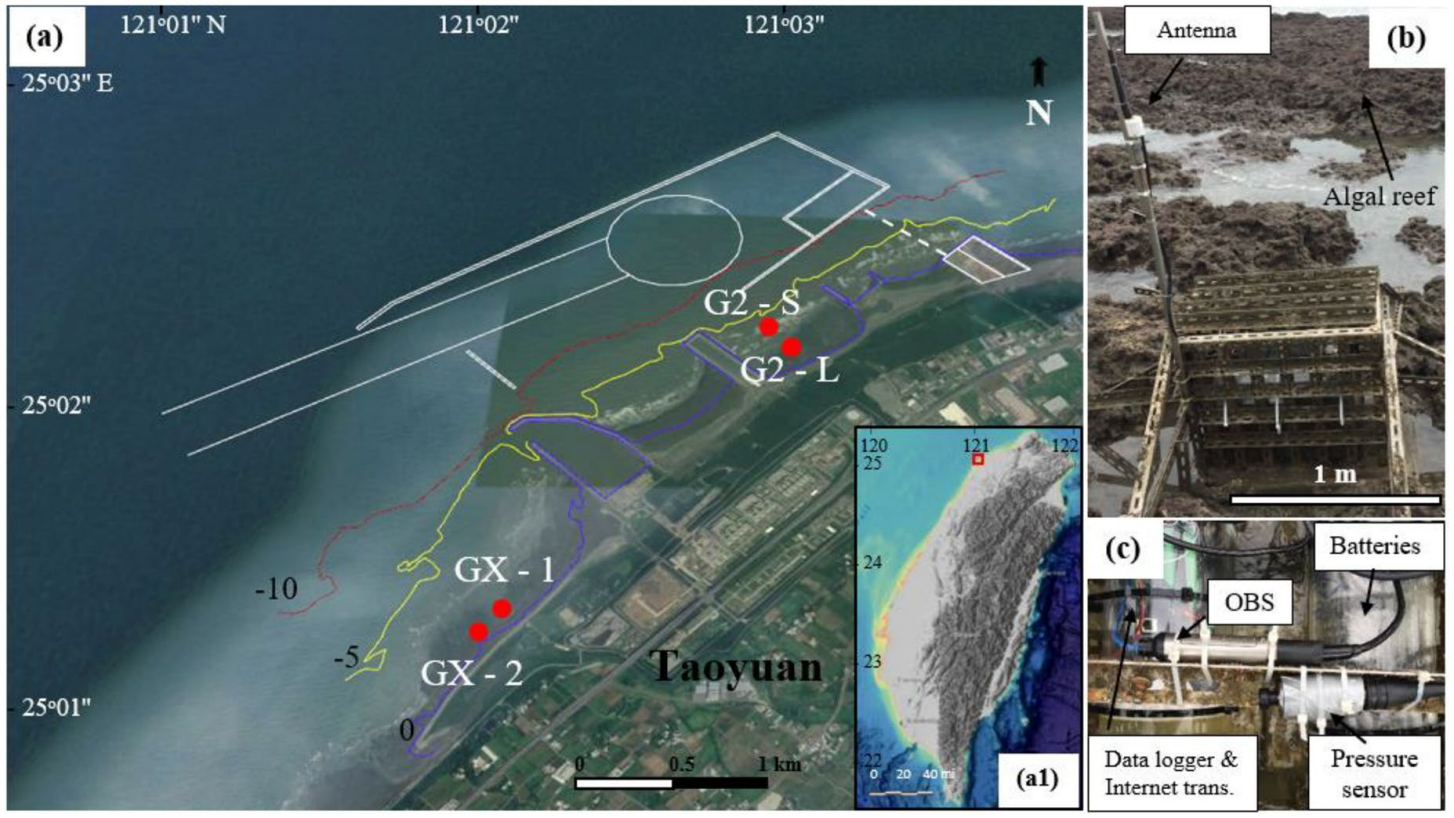
**a** The location of the Taoyuan coast with the sites of the instruments relevant to this study (red dots) and contours indicated for water depth. Figure (a1) shows the Taiwan map; the red square indicates the study location. The white lines in **a** indicate the construction of the LNG receiving port. **b** Picture of the instrument station at site G2-S. The 3-m antenna helps transmit data during high tide levels. **c** A picture of the instruments inside the monitoring system

A unique algal reef ecosystem was found along the Taoyuan coast, Taiwan, which is considered to have unique endemic species populations (Liou et al., 2017). A liquefied natural gas (LNG) receiving port project (white lines in Fig. 1) was launched in 2019 near the reef ecosystem because of its intended energy security and essential economic benefits (Chen et al., 2019). Coastal construction raises some environmental issues for this sensitive ecological area. The discovery of a newly described endemic coral species, *Polycanthus chaishanensis*, and changes in the compositional layers over time highlight the importance of this unique ecosystem in Taiwan (Kuo et al., 2020). Not only do dredging operations impact the reef system during construction activities, but the changing hydrodynamic and sediment dynamics due to construction might also affect the ecosystem (Fatahi Nafchi et al., 2021; Ostad-Ali-Askari et al., 2017). Monitoring changes in sediment and hydrodynamic processes is therefore very important for the preservation of coastal habitats.

Several studies have confirmed that *c* is a major detrimental factor affecting reef health (Jones et al., 2020; Ogston et al., 2004). Both high and low *c* values can adversely affect various benthic reef organisms (Pomeroy et al., 2017). An increased sediment transport rate prevents light penetration into coral reefs, weakening reef community health (Storlazzi et al., 2015). The overly critical *c* could lead to reef system mortality (Jokiel et al., 2014). Simultaneously, a low or lacking *c* can impact several critical ecological processes on reefs. Sediment can provide a vital ecological function on reefs, such as infilling crevices, cementing the reef framework, suppressing herbivory (Goatley, 2013), or helping corals obtain nutrients, such as suspended particulate matter (Kuo et al., 2020). The spatial and temporal variability in *c* might significantly affect the distribution of species in reefs (Kuo et al., 2020). However, research on these topics is still limited, particularly in ecosystems that are vulnerable to environmental changes.

Most studies pay attention to *c* in coral reef ecosystems. The reported *c* ranges from 3 to 40 mg/l (Bell, 1990; Hoitink, 2003, 2004; Larcombe et al., 2001; Pomeroy et al., 2017, 2018; Roy & Smith, 1971), and sometimes, a higher *c* was also found in other coral reefs, with a range from 75 to 260 mg/l (Hopley et al., 1993; Mapstone et al., 1992; Rice & Hunter, 1992). However, the range, viability of *c*, and major factors driving *c* are not clear in an algal reef environment. Noticeably, there are few long-term monitoring studies of c in reef systems, especially studies exploring the change in *c* in different seasons and coupling with hydrodynamic measurements. Little is known about the algal reef system compared to other abundant studies on coral reef systems (Liou et al., 2017). The values and changes in *c* in algal reef environments are not well understood.

Many studies in recent years have focused on the short-term or long-term monitoring of *c* but without continuous data (Adamo et al., 2015; Buschmann et al., 2012; Robert et al., 2016). The traditional method for monitoring *c* is to collect and analyze water samples. The advantage of this method is that it is easy to interpret and independent of factors such as grain size, shape, and concentration (Nielsen, 1992). However, this method is not suitable for continuous and long-term studies on the monitoring of *c* due to difficulties in collecting water samples during typhoons and limited resources. To capture the instantaneous *c*, an optical backscatter sensor (OBS) has been developed because of its small size, low power, highly linear response, and insensitivity to bubbles or organic matter. As a result, this method has been applied to measure *c* (Felix et al., 2018; Hoitink & Hoekstra, 2005; Lu et al., 2017; Pang et al., 2020; Pomeroy et al., 2017, 2018; Ruhl & Schoellhamer, 2004; Schoellhamer, 2002). An OBS has been widely used, but other factors, such as sediment size and concentration, which vary with space, affect the sensor response and lead to incorrect measurements of *c* (Cuttler et al., 2017; Downing, 2006; Pomeroy et al., 2017). An OBS is not a means to directly measure *c*, and calibration is necessary for OBS use. An OBS can be applied to the long-term monitoring of *c*; however, the accuracy of the OBS measurement and the number of calibrations remain questions when applying this instrument for monitoring on a year-long time scale.

We present and assess a monitoring system for the real-time monitoring of waves and *c* in a shallow algal reef area. The aims of this study are as follows: (i) describe and examine the accuracy of these monitoring stations over year-long timescales, and (ii) understand the significant drivers of *c* in this reef environment. The long-term data will enable an assessment of how the construction of LNG infrastructure will affect *c* in the near future. The monitoring system, including an OBS and a pressure sensor, was integrated with a data logger and a real-time internet transmission system. Section 2 presents the methodologies, including the monitoring system equipment and deployment in the field. In addition, the results of the testing calibration OBS and the relationship between the hydrodynamics and *c* are discussed in Sect. 3. Finally, the conclusion is presented in the final section.

## Methodology

### Site descriptions and field measurements

The study area is located in the intertidal zone on the coast of Taoyuan, northern Taiwan (121° 3′3.86″ N, 25° 2′18.93″ E) (Fig. 1a). Most of the intertidal zone is an abiotic reef, and only a small part is covered with sand and cobbles. The biotic reef flat extends approximately 27 km alongshore with a maximum width of 500 m and an average slope of 0.7%. The reef is primarily composed of crustose coralline algal (CCA) concretions or algal reefs (Liu et al., 2018). However, algal reefs have been threatened by industrial pollution and coastal construction (Liou et al., 2017). Figure 1a depicts the existing construction of the jetties and the planned LNG harbor.

Four study sites were selected for the long-term monitoring of *c* over the reef. These sites were classified into two groups: one in the Guanxin Algal Reefs Ecosystem Wildlife Refuge (denoted as GX) and the other in the Datan reef area (denoted as G2). The acronyms are summarized in the Appendix. The two areas of the first group were designated GX-1 (25°01′09.64″ N, 121°01′56.74″ E) and GX-2 (25°01′6.486″ N, 121°01′54.928″ E) and were located in the Refuge, which was outside the LNG harbor construction area; they were separated by a distance of 190 m alongshore under similar water depths. The two areas of the second group were designated G2-S (25°02′16.82″ N, 121°02′52.10″ E) and G2-L (25°02′12.12″ N, 121°02′56.13″ E), which were located in Datan; they were separated by 180 m in the cross-shore direction (Fig. 1a).

Detailed deployment time and sampling information for each instrument are shown in Table 1. Both the OBS and the pressure sensors were placed approximately 0.49 m above the reef bottom. Moreover, acoustic Doppler profilers (ADPs) were deployed near site G2-S to measure the waves and currents during the typhoon period. However, the ADP showed inferior quality; therefore, it provided only significant wave height (*Hs*). Consequently, no current directions or velocities were obtained in the results. In this paper, we present a 1-year monitoring dataset from 19 July 2019 to 19 July 2020. This period was selected because it included a typhoon that occurred in August 2019.

**Table 1:** Instrument site information and sampling configuration

Location	Average depth (m)	Instrument	Settings	Measurements	Time period
G2-L	1.5	OBS	SR = 2 Hz, recording for the first 2 min every hour	Turbidity	01/01/2019–present
		Pressure sensor	SR = 4 Hz, continuous measurement	Wave elevation, tide	27/10/2019–present
G2-S	3.5	OBS	SR^a^ = 2 Hz, recording for the first 2 min every hour	Turbidity	19/07/2019–present
		Pressure sensor	SR = 4 Hz, continuous measurement	Wave elevation, tide	19/07/2019–18/08/2021
		Acoustic Doppler current profiler (ADP)	Measure current profiles at a 1 Hz with an averaging interval of 2 min	Velocity profile every 30 min (Failed) Significant wave height every 30 min	18/07/2019–15/8/2019
GX-1	1.6	OBS	SR = 2 Hz, recording for the first 2 min every hour	Turbidity	01/01/2019–present
GX-2	1.6	OBS	SR = 2 Hz, recording for the first 2 min every hour	Turbidity	01/01/2020–present

^a^*SR* Sampling Rate

### The real-time monitoring system

We used a monitoring system to conduct real-time, continuous, automatic observations of waves and *c*. The real-time monitoring system included three main components: (i) OBS for measuring the *c* and pressure sensor for measuring the waves; (ii) data logger for recording the data; and (iii) real-time internet transmission system (MOXA), which was connected with a 3-m-high antenna (Fig. 1b) to obtain the real-time measurements. The data were transmitted over a fourth-generation broadband cellular network technology (4G) wireless communication system to the host computer. This MOXA was fitted to go online within 10 min every 2 h to receive the new program or transmit the new data; this process helped save power for the whole system. The power of all three components was supplied by a battery tank, a combination of two individual 19.40-V batteries (tank in Fig. 1c). The battery tank could supply power for this system for more than one month; however, we set a safe cycle of 28 days to replace the battery tank. The system was deployed in a steel box with dimensions of 60-cm width, 100-cm length, and 55-cm height to protect the instruments from damage and theft (Fig. 1b).

**Fig. 2:**
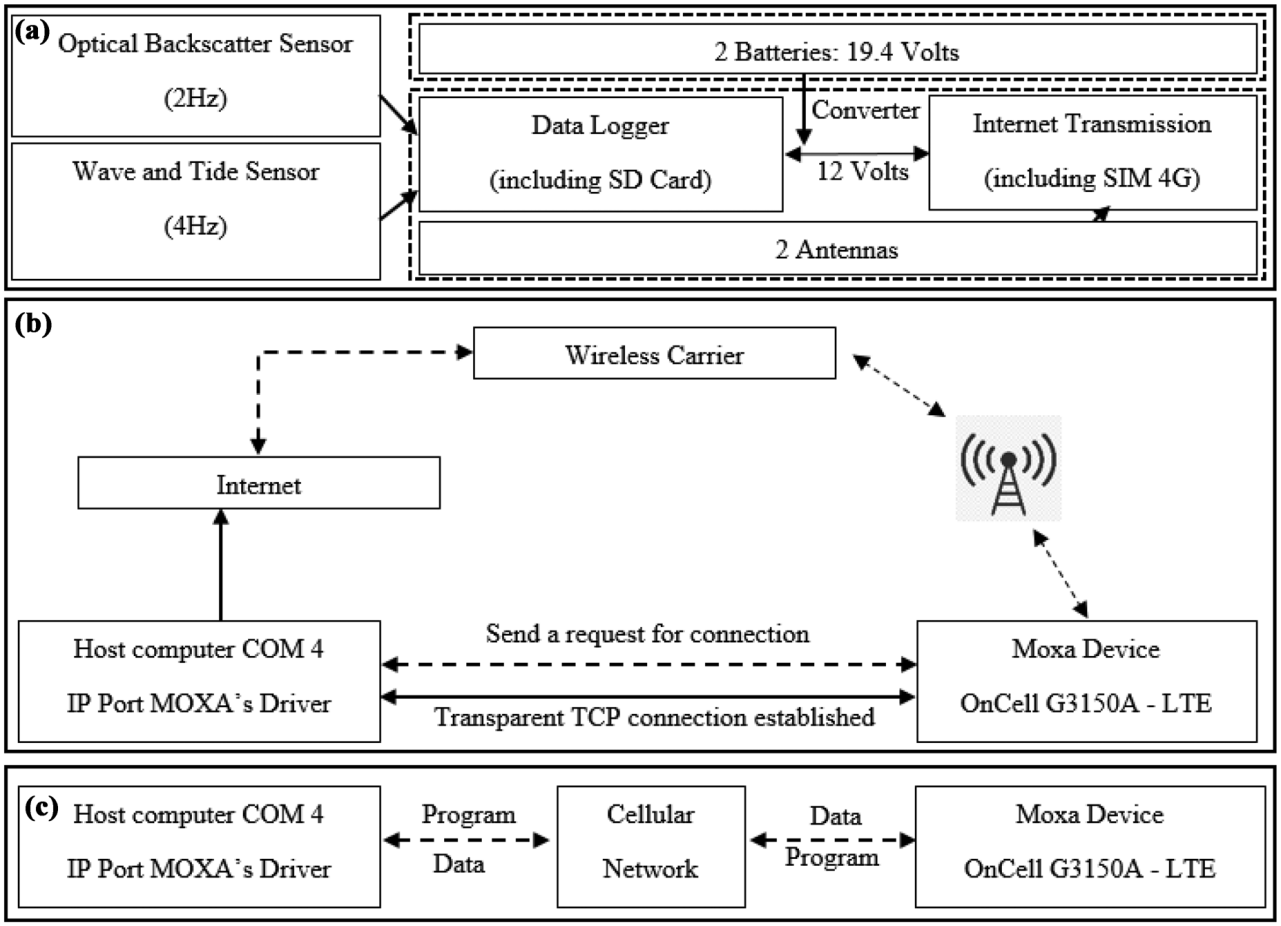
**a** A system for data acquisition flowchart. The dashed line indicates the waterproof tank, which contains all the batteries and instruments. **b** Schematic illustration of the real communication connection. **c** Host computers send programs and receive data and vice versa for MOXA

The components of the monitoring system and data acquisitions are presented in Fig. 2. All measured data from the OBS, pressure sensors, and timestamps were recorded on a secure digital card (Fig. 2a). Additionally, this system allowed us to change the sampling configuration remotely. As illustrated in Fig. 2b, data transmission proceeded as follows: The host computer requested a connection from the on-cell, which was configured for server mode. Once the connection was established, the OBS and pressure data could be transmitted in both directions between the host computer and MOXA. After connecting to the cellular network, the service provider offered a dynamic public internet protocol (IP) address and then accessed the MOXA from a host computer using a fixed public IP (Fig. 2c). The system was applied to monitor *c* in the conservation zone where algal reef damage must be restricted. It also helped solve several problems involved in monitoring *c* in the intertidal zone due to the long distances (Adamo et al., 2015). As a result, this monitoring method proved to be an efficient and low-impact instrument for reef monitoring.

### Data analysis

#### Hydrodynamic conditions

**Fig. 3:**
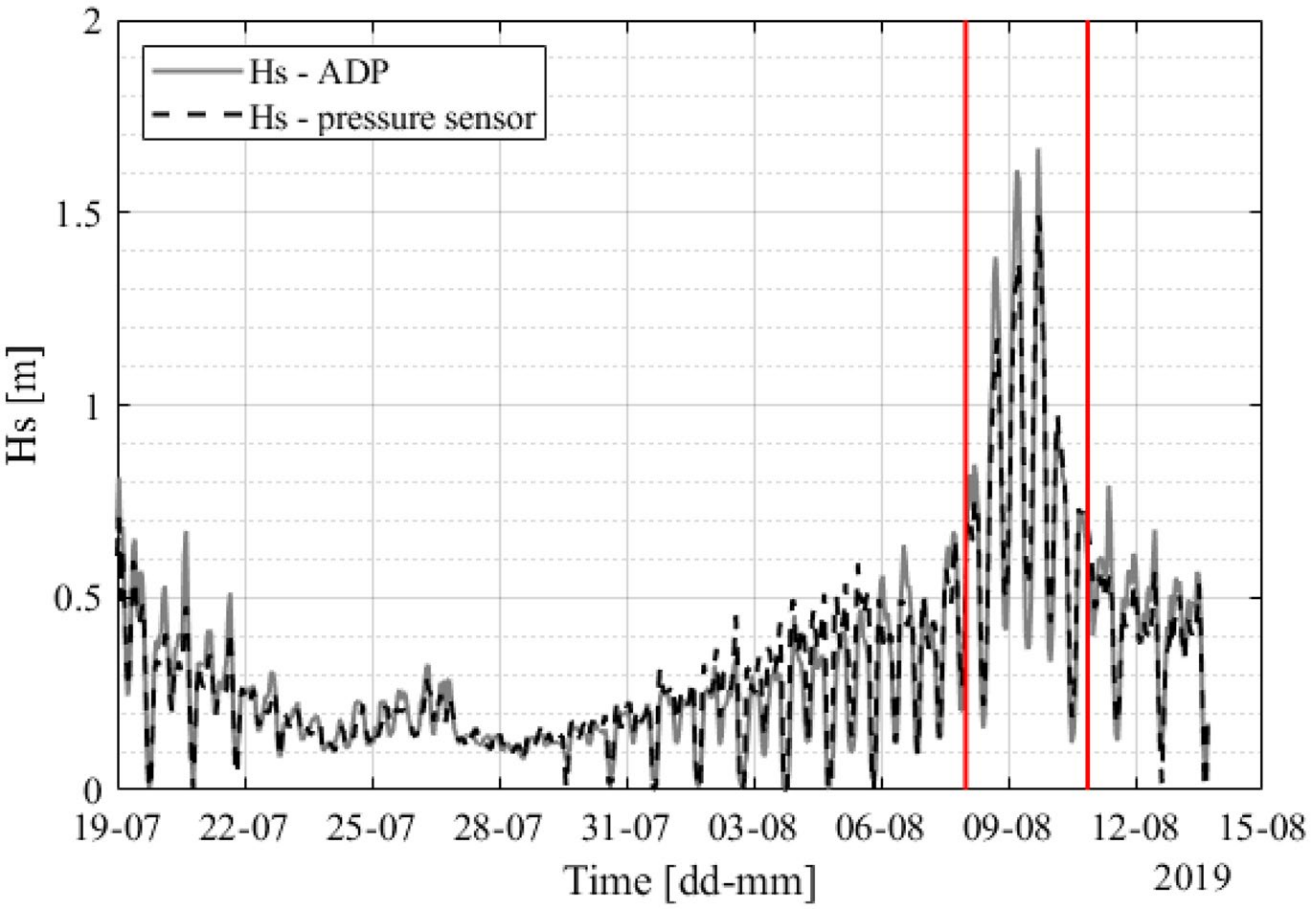
The comparison of *Hs* was measured by an ADP (gray line), and *Hs* was computed by Eq. (1) from the pressure sensor data (dashed black line). The red vertical lines indicate the *Hs* during the typhoon period

We installed a pressure sensor in the real-time monitoring system to measure wave properties at the same location as the OBS. The significant wave height was computed from the pressure data. The definition of significant wave height, *Hs*, is as Eq. (1) (Young, 1999):

1 $$Hs=4{m}_{0}=4{\left[\int {S}_{\upeta }\left(f\right)df\right]}^{1/2}$$

where ${m}_{0}$ is the variance of the water surface elevation, *η*, and ${S}_{\eta }(f)$ is the spectral density of surface displacement as a function of frequency, *f*. Significant wave height is computed as the integral of the surface spectrum obtained from the pressure spectrum with linear wave theory. The significant wave height measured by the pressure sensor is compared with *Hs* measured by the ADP. Good agreement was shown, even for strong wave conditions during the typhoon (Fig. 3).

Linear wave theory and spectral analysis were used to calculate the root mean square (*RMS*) near-bottom horizontal velocity ($${u}_{b})$$ (Ole Secher, 1994) as follows:

2 $${u}_{b}=\sqrt{\sum_{n=1}^{N}{u}_{b,n}^{2}} ,$$

where ${u}_{b,n}$ is the near-bottom horizontal orbital velocity of the n^th^ component:

3 $${u}_{b,n}=\frac{{a}_{n}{\omega }_{n}}{sinh{k}_{n}h} ,$$

where ${\omega }_{n}$ and ${a}_{n}$ are the radian wave frequency and amplitude of the n^th^ component, respectively. ${a}_{n}$ is determined by the following:

4 $${a}_{n}= \sqrt{2{S}_{n}df} .$$

The bottom stress (*τ*) for wave-current flows was combined with the current shear stress (${\tau }_{c}$) and the wave-bed stress (${\tau }_{w})$ (Jia et al., 2014). Because the effect of wave motion-induced shear stress on *c* should be dominant in the shallow algal reef (Pomeroy et al., 2017; Wiberg & Sherwood, 2008), we first ignored the bed stress induced by the current and considered the wave bed stress (${\tau }_{w}$) as follows:

5 $${\tau }_{w}={\rho {u}_{*}^{2}}_{w}=\frac{1}{2}\rho {f}_{w,r}{u}_{b}^{2}$$

where *ρ* is the water density and the wave-bed stress is based on wave-induced ${u}_{*w}$ (shear velocity).

The wave friction factor, ${f}_{w,r}$, is an essential dimensionless parameter used to estimate wave-induced bed shear stress (Le Roux, 2003, 2012; Lu et al., 2017). Several formulas have been developed to predict the wave friction factors under wave conditions (Ivar, 1966; Le Roux, 2012; Ole Secher, 1994; Smyth & Hay, 2002; Swart, 1974). In addition, the wave friction factor has been recognized as a function of the near-bed flow velocity and seabed roughness (Nielsen, 1992). The empirical calculations of ${f}_{w,r}$ were proposed by Swart (1974) in the following form:

6 $${f}_{w,r}=exp\left[{c}_{1}{\left(\frac{{u}_{b}}{{k}_{w}{\omega }_{r}}\right)}^{{c}_{2}}+{c}_{3}\right] ,$$

where ${\omega }_{r}$ is the representative radian frequency in the form of

7 $${\omega }_{r}=(\sum_{n=1}^{N}{\omega }_{n}{u}_{b,n}^{2})/(\sum_{n=1}^{N}{u}_{b,n}^{2})$$

We used the constants ${c}_{1}$*,* ${c}_{2}$, and ${c}_{3}$ at values of 7.02, − 0.078, and − 8.82, respectively, which were proposed previously (Mathisen & Madsen, 1999; Ole Secher, 1994). These values are suitable for spectral wave conditions, and a rough bottom surface instead of the coefficient is fitted in the laboratory for monochromatic waves (Nielsen, 1992). ${k}_{w}$ is the Nikuradse roughness in the representation of the bottom roughness and is defined as four times the single RMS length scale of the bottom elevation (${\sigma }_{b})$ Huang et al., 2012; Lowe et al., 2005; Nielsen, 1992):

8 $${k}_{w}=4\cdot {\sigma }_{b}$$

The bottom roughness of the algal reef was measured in situ by the UAV-RTK Lidar System developed by Huang et al. (2018) (not shown here for brevity). The single length standard deviation of elevations with topography removal was 0.19 m, and ${k}_{w}$ = 0.76 m. This algal bottom roughness was higher than the coral reef bottom roughness, which was ${\sigma }_{b}$ = 0.14 m, but ${k}_{w}$ = 1.09 m because of ${k}_{w}=4{\mathrm{H}}_{\mathrm{bed}}$ (Ole Secher, 1994), which was better than ${k}_{w}=4\cdot {\upsigma }_{\mathrm{b}}$ (Huang et al., 2012; Nielsen, 1992). The bottom roughness of the barrier reef, ${k}_{w}$ = 0.16 m with ${\upsigma }_{\mathrm{b}}$ = 0.036 m (Lowe et al., 2005), was also lower than the bottom roughness in this study.

#### Suspended sediment concentrations (c)

OBSs were installed in the real-time monitoring systems to measure *c*. Because the OBS does not directly measure *c*, a calibration process is needed for the OBS. More details of the OBS calibration process are presented in the following. *c* was inferred from four OBS samplings at 2 Hz (Table 1) and was compared with a water sample analysis. The water samples were collected every 2 weeks in tanks (4 L) deployed at sites G2-L and GX-1. The tank had unique designs in that they were filled with water from the top and then sealed tightly automatically. The filled tanks were divided into two subsamples and shipped directly to the laboratory. One subsample was analyzed in our lab. It was vacuum filtered (What-man M27, 8 µm), dried (105 °C for 1 h), and weighed to calculate *c*. After filtration, the filters were cleaned with pure water to remove salts. Another subsample was directly shipped trimonthly to the Environmental Technology Research Center, Yuan Ze University, where they were analyzed using standard methods that were suitably adjusted to handle a small quantity of water sample. The results analyzed from different groups were consistent (Fig. 7).

**Fig. 4:**
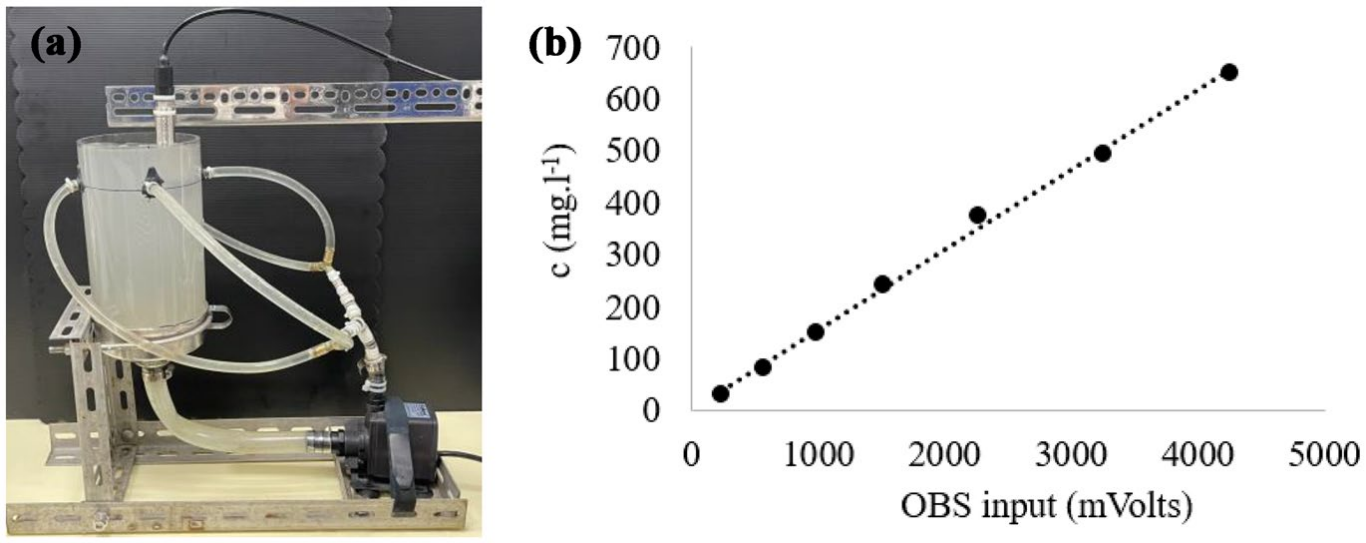
**a** The specially designed tank was used for calibrating the OBS, which helped to mix the water samples well. This system ensured the homogeneity of the water samples. **b** An example of a calibration curve from the linear regression of the OBS output and *c*. The results show a robust linear regression of the OBS output and *c*

The OBS was calibrated by using a specially designed tank system. The tank was designed to ensure that the suspended sediment in the water was uniformly distributed, leading c to not be a function of the spatial position (Fig. 4a). Therefore, it helped to minimize the error between the water sample and subsample during the OBS calibration process. The variation among the samples was negligible. As a result, the robust linear regression of the *c* measurements taken from water samples against the OBS voltage is shown in Fig. 4b.

#### Singular-spectrum analysis

To evaluate the *c* variability over a long timescale, the singular-spectrum analysis (SSA) technique was applied (Golyandina & Zhigljavsky, 2013; Pomeroy et al., 2018; Schoellhamer, 2002; Wang et al., 2019). The daily averaged time series data of *c* were then analyzed with the SSA technique. This technique identifies a set of uncorrelated time-dependent variables that describe different fractions of the original signal variance. We selected two principal components, mode 1 and mode 2, representing seasonal and spring-neap tidal cycle components, respectively. The dominant advantage of this method is that it is nonparametric and can trend periodic or quasiperiodic components of the time-series data (Pomeroy et al., 2018). In this analysis, we used a window period of M = 14 days because this period was dominated by the frequency from the FFT analysis.

## Results

### Hydrodynamic conditions

**Fig. 5:**
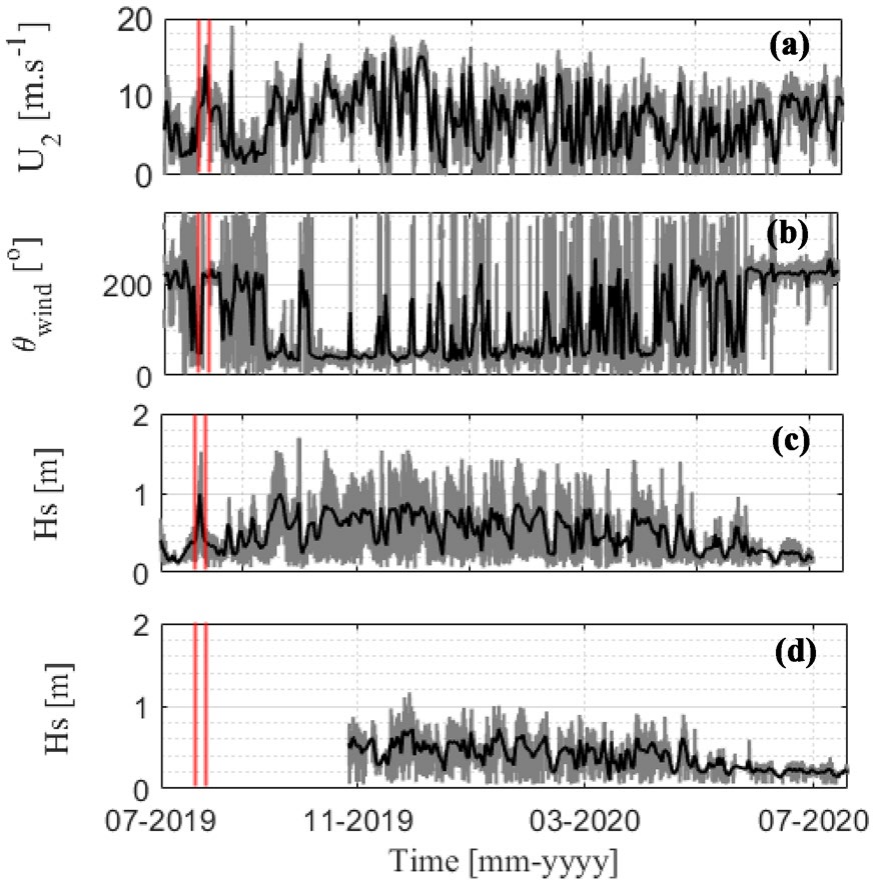
Time series of wind conditions and *Hs*. **a** Wind speed and **b** wind direction collected from the Central Weather Bureau, Taiwan. Panels **c** and **d** show the time series of *Hs* at sites G2-S and G2-L, respectively. The gray and black lines represent the hourly average and daily average data, respectively. The red vertical lines indicate the typhoon period. The missing data in **c** are due to the failure of pressure sensors

The time series of the wind properties during the entire monitoring year is shown in Fig. 5a and b. The wind speed was relatively weak during the summer season (6.4 m/s), and it became stronger during the winter season (7.3 m/s), except for the rapidly increased velocity during the typhoon period (10.8 m/s). Similarly, the wind direction ranged from 189 to 250 degrees during summer and 18 to 35 degrees during winter. Overall, the winds tended to be stronger during the winter season and were relatively weak during the summer season; however, the wind increased with the arrival of the typhoon event during the middle of August.

The tidal regime on the Taoyuan coast showed a semidiurnal tide. The tidal range at site G2-S varied from approximately 0 to 2.2 m during neap tide, while it varied from 0 to 3.5 m during spring tide. In addition, the water depth at site G2-L varied with a smaller range; it was approximately 0 to 1.5 during neap tide, and it was approximately 0 to 2.8 m during spring tide. The water depth at site G2-S was higher than that at site G2-L due to the bottom slope condition.

The time series of *Hs* varied similarly to the wind speed trend, which was higher during winter and smaller during summer (Fig. 5c and d). During the summer season of 2019, *Hs* was small and relatively constant, ranging from 0.4 to 0.8 m at site G2-S (no data for site G2-L). However, *Hs* rapidly increased in early August 2019 (*Hs* = 1.71 m) due to the typhoon event and quickly decreased after the typhoon passed (Fig. 5c). In addition, *Hs* ranged from 0.6 to 1.3 m at site G2-S during the winter season. Overall, the *Hs* measured at site G2-S was correlated (*R*^*2*^ = 0.69) but was more than 1.3 times higher at site G2-L. The limitation of the water depth explained for *Hs* at site G2-L was not strongly modulated, ranging from only 0.2 to 0.8 m. As a result, the smaller *Hs* at site G2-L than that at site G2-S was reasonable due to the depth-limited wave breaking and bottom friction (Huang et al., 2012; Pomeroy et al., 2017, 2018).

**Fig. 6:**
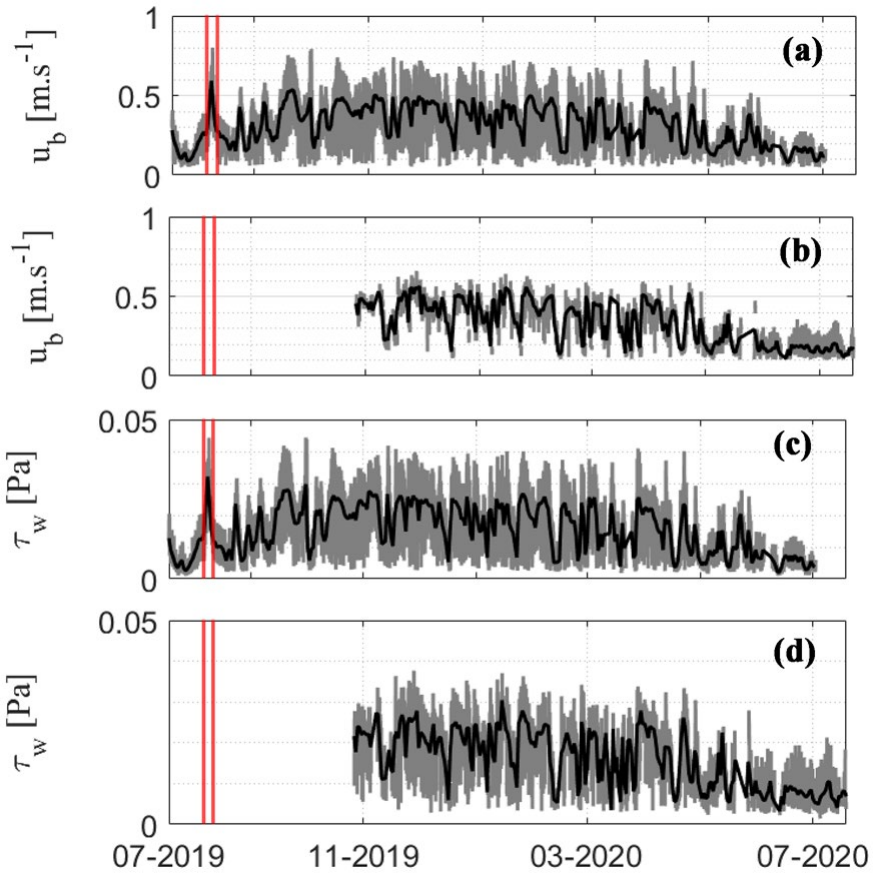
Near-bottom horizontal orbital velocity (${u}_{b}$) at sites (a) G2-S and (b) G2-L. The two bottom panels show the estimated wave-bed stress (${\tau }_{w}$) of sites (c) G2-S and (d) G2-L. The gray and black lines represent hourly average data and daily average data, respectively. The red vertical lines indicate the typhoon period

The time series of near-bottom horizontal orbital velocity ($${u}_{b})$$ for the entire monitoring year is presented in Fig. 6a and b. Similar to the trend of *Hs*, the estimated $${u}_{b}$$ during winter (0.4 to 0.7 m/s) was higher than the $${u}_{b}$$ values during summer (0.1 to 0.4 m/s) at site G2-S (Fig. 6a). At site G2-L, the estimated $${u}_{b}$$ was almost comparable, ranging from 0.3 to 0.6 m/s and from 0.1 to 0.3 m/s during winter and summer, respectively (Fig. 6b). The larger significant wave event in winter caused the offshore waves to become prominent, increasing the magnitude of the $${u}_{b}$$ at all sites. Overall, the daily average $${u}_{b}$$ was higher during winter and substantially smaller during summer. During the winter season, $${u}_{b}$$ increased slightly onshore (G2-L) compared with offshore (G2-S) due to the limitation of the water depth. Waves in shallow water move in ellipses and subsequently grow flatter toward the bottom, indicating that the vertical orbital velocities degenerated faster than the horizontal velocity (Bian et al., 2020). The variation in $${u}_{b}$$ showed a different trend between the two seasons when applying the SSA. The temporal decomposition of the daily average $${u}_{b}$$ at site G2-S revealed that 93.98% of the variance in $${u}_{b}$$ was described by the seasonal mode (mode 1) (Table 2). A further 1.64% of the $${u}_{b}$$ variance occurred due to the spring-neap tidal regime (Mode 2). A similar mode of $${u}_{b}$$ variability was also measured at site G2-L, where 95.6% of the variability was explained by the two modes, consisting of the dominant mode 1 (94.09% due to seasonal variation) and mode 2 (1.41% due to spring-neap tidal regime) (Table 2).

**Table 2:** Singular-spectrum analysis (SSA) of ${u}_{b}$, ${\tau }_{w}$, and *c* at the four monitoring sites (units for modes 1 and 2 are %)

Site	${u}_{b}$		${\tau }_{w}$		*c*		${u}_{b}$ vs. *c*^a^		${\tau }_{w}$ vs. *c*^b^	
	Mode 1	Mode 2	Mode 1	Mode 2	Mode 1	Mode 2	Reg.^c^ 1	Reg.^d^ 2	Reg. 1	Reg. 2
G2-S	93.98	1.64	91.22	2.26	76.41	7.00	0.56	0.49	0.57	0.49
G2-L	94.09	1.41	93.93	1.76	78.51	7.69	0.85	0.53	0.83	0.47
GX-1	-	-	-	-	81.26	7.93	-	-	-	-
GX-2	-	-	-	-	77.54	5.27	-	-	-	-

^a^A goodness-of-fit measure for linear regression models (R^2^) between two modes of 𝑢_𝑏_ vs c

^b^A goodness-of-fit measure for linear regression models (R^2^) between two modes of $\tau_w$  vs c

^c^Reg. 1: Linear regression between Mode 1 of 𝑢_b_ and Mode 1 of c

^d^Reg. 2: Linear regression between Mode 2 of 𝑢_𝑏_ and Mode 2 of c

^e^-: no data available at two monitoring sites (GX-1 and GX-2)

The time series data of the daily average wave-induced bed stress (${\tau }_{w})$ for the whole monitoring year are presented in Fig. 6c and d. The ${\tau }_{w}$ was high during the typhoon event at site G2-S (${\tau }_{w}$= 0.032 Pa) (no data at site G2-L). Overall, the ${\tau }_{w}$ was larger at the offshore site (G2-S) and smaller at the onshore site (G2-L). The SSA showed the temporal decomposition of ${\tau }_{w}$ due to seasonal and spring-neap tidal variations (Table 2). Mode 1 revealed that 91.22% of the variance in the ${\tau }_{w}$ was described by the seasonal variation at site G2-S. An additional 2.26% of the ${\tau }_{w}$ variance occurred due to the spring-neap tidal regime. Similarly, the ${\tau }_{w}$ variability was estimated at site G2-L, where 93.93% of the variability was explained by two modes, consisting of 92.17% from the season (mode 1) and 1.76% from the tidal regime (mode 2). From the average ${\tau }_{w}$ of mode 1, the variation trend clearly showed a higher ${\tau }_{w}$ during the winter season than during the summer season.

### Suspended sediment concentrations

#### Testing of the calibration value of the OBS

Because the OBS was not a direct measurement of *c*, a detailed calibration was needed for OBS measurement. The detailed calibration values of the OBS are presented in Table 3. There were 13 cases of testing OBS calibration, from applying one calibration value (T1–T4) to four calibration values following four seasons (T13) for the whole year of *c* measurement. For instance, in the first case (T1), we applied only one linear equation obtained during summer to calibrate the OBS for the whole-year period. Four calibration values of the OBS (case T13) were applied following the four seasons for the whole-year monitoring. The root mean square error (RMSE) from the testing regression of *c* obtained from the OBS and water sampling is shown in Table 3. Test case T1 had the highest RMSE, which was approximately 61.43 mg/l, followed by that of case T3, with an RMSE of approximately 60.88 mg/l. Using only one calibration value for the whole-year measurement, test case T2 had a small RMSE value (44.91 mg/l). The smallest RMSE (43.02 mg/l) was primarily found after applying four calibration values following four seasons (T13). Overall, this testing showed that the RMSE values deviated slightly from 43 to 61 and could be reduced by increasing the calibration time for the OBS measurement.

**Table 3:** Testing of OBS calibration value. Different calibration equations were obtained in different seasons to test the *c* values measured by the OBS and by water sampling

Testing cases	Summer (Jun.–Aug.)	Fall (Sep.–Nov.)	Winter (Dec.–Feb.)	Spring (Mar.–May.)	*RMSE*^a^	*SE*^b^	Slope^c^	*R*^*2*^^d^
	Equation (1): *c* = 0.2162x—20.19	Equation (2): *c* = 0.2495x—16.35	Equation (3): *c* = 0.3037x—14.15	Equation (4): *c* = 0.2302x—13.28				
T1	Equation (1)				61.43	19.95	0.78	0.87
T2	Equation (2)				44.91	23.02	0.92	0.87
T3	Equation (3)				60.88	28.01	1.07	0.86
T4	Equation (4)				49.85	21.24	0.86	0.87
T5	Equation (1)		Equation (3)		56.09	24.96	0.95	0.80
T6	Equation (1)		Equation (4)		55.31	20.49	0.82	0.87
T7	Equation (2)		Equation (3)		49.40	25.73	1.03	0.85
T8	Equation (2)		Equation (4)		48.52	22.23	0.89	0.85
T9	Equation (1)	Equation (2)	Equation (3)		46.73	25.79	1.02	0.86
T10	Equation (1)	Equation (2)	Equation (4)		45.79	22.05	0.88	0.89
T11	Equation (1)		Equation (3)	Equation (4)	52.50	24.54	0.92	0.83
T12	Equation (2)		Equation (3)	Equation (4)	45.00	25.59	0.98	0.87
T13	Equation (1)	Equation (2)	Equation (3)	Equation (4)	43.02	25.74	0.99	0.88

^a^Root mean square error (RMSE)

^b^Standard error of the OBS data (SE)

^c^Slope in the linear equation between OBS data and water sampling data (Slope)

^d^A goodness-of-fit measure for linear regression models (R^2^)

The linear regression slopes of *c* between the water sample analysis and OBS data are illustrated in Table 3. These slopes were lower during summer and higher during winter. The different values of the regression slope might be due to the changes in sediment particle size and concentration (Black & Rosenberg, 1994; Cuttler et al., 2017; Downing, 2006; Felix et al., 2018; Lynch et al., 1994; Pomeroy et al., 2017, 2018; Rai & Kumar, 2015; Ruhl & Schoellhamer, 2004; Schoellhamer, 2002). Applying only one calibration equation (cases of T1, T2, T3, and T4) for the entire monitoring year of *c*, the linear regression slopes were 0.78, 0.92, 1.07, and 0.86, respectively. A slope larger than 1 indicates an overestimate of *c* for the OBS measurements. An overestimated *c* was found when applying the calibration equation during winter (T3), with an RMSE value = 60.88 mg/l. Corresponding to the coefficient value, a higher slope was found during winter, possibly due to the larger particle size and denser sediment concentration (Downing, 2006; Felix et al., 2018; Lynch et al., 1994). The best regression slope was found when using four calibration equations (case T13) with a linear regression slope of 0.99.

Overall, this sensitivity testing on the calibration equations demonstrated that for the long-term monitoring of *c*, the OBS should be calibrated seasonally for the best comparison between OBS data and water sampling. The finding also suggested that there might be particle-size variation among seasons and might produce under- or overestimates in *c* that are unrelated to real changes in the sediment concentration. A greater number of OBS calibrations leads to a higher accuracy of the *c* measurement using OBS. However, the testing results showed that the regression coefficients (0.86–0.87) and RMSE (50–61) were similar if we calibrated the OBS only once (T1 to T4). The slope became 0.78 to 0.8 for some cases, indicating that we should be careful about extrapolating the calibration equation. The test cases from T1 to T4 showed that the calibration values obtained during either the summer or winter (T1 or T3) had larger RMSEs than those of other seasons. Cases T2 and T4 showed a better slope and a smaller RMSE. These findings suggest that the calibration of the OBS in the spring and fall seasons is a compromise with an equivalent error when handling just one calibration value for whole-year monitoring.

### Variation in suspended sediment concentrations

**Fig. 7:**
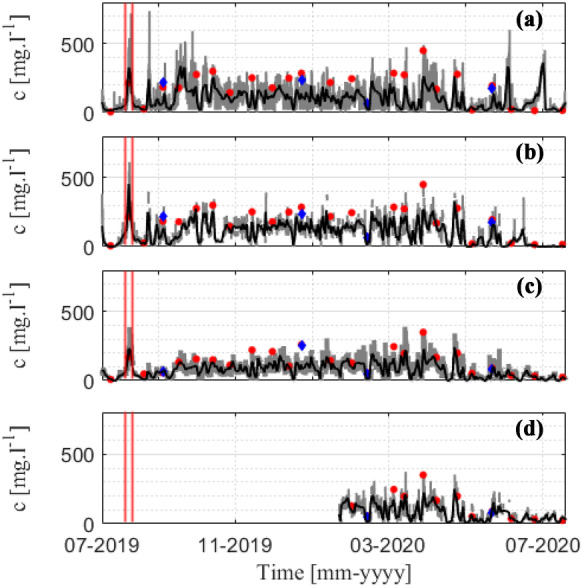
Time series of the measured *c* at the four monitoring sites: **a** G2-S, **b** G2-L, **c** GX-1, and **d** GX-2. The gray and black lines represent the hourly average and the daily average data, respectively. The red dots and blue squares indicate *c* from water sampling in our laboratory and another laboratory, respectively. The red vertical lines indicate the typhoon period

**Fig. 8:**
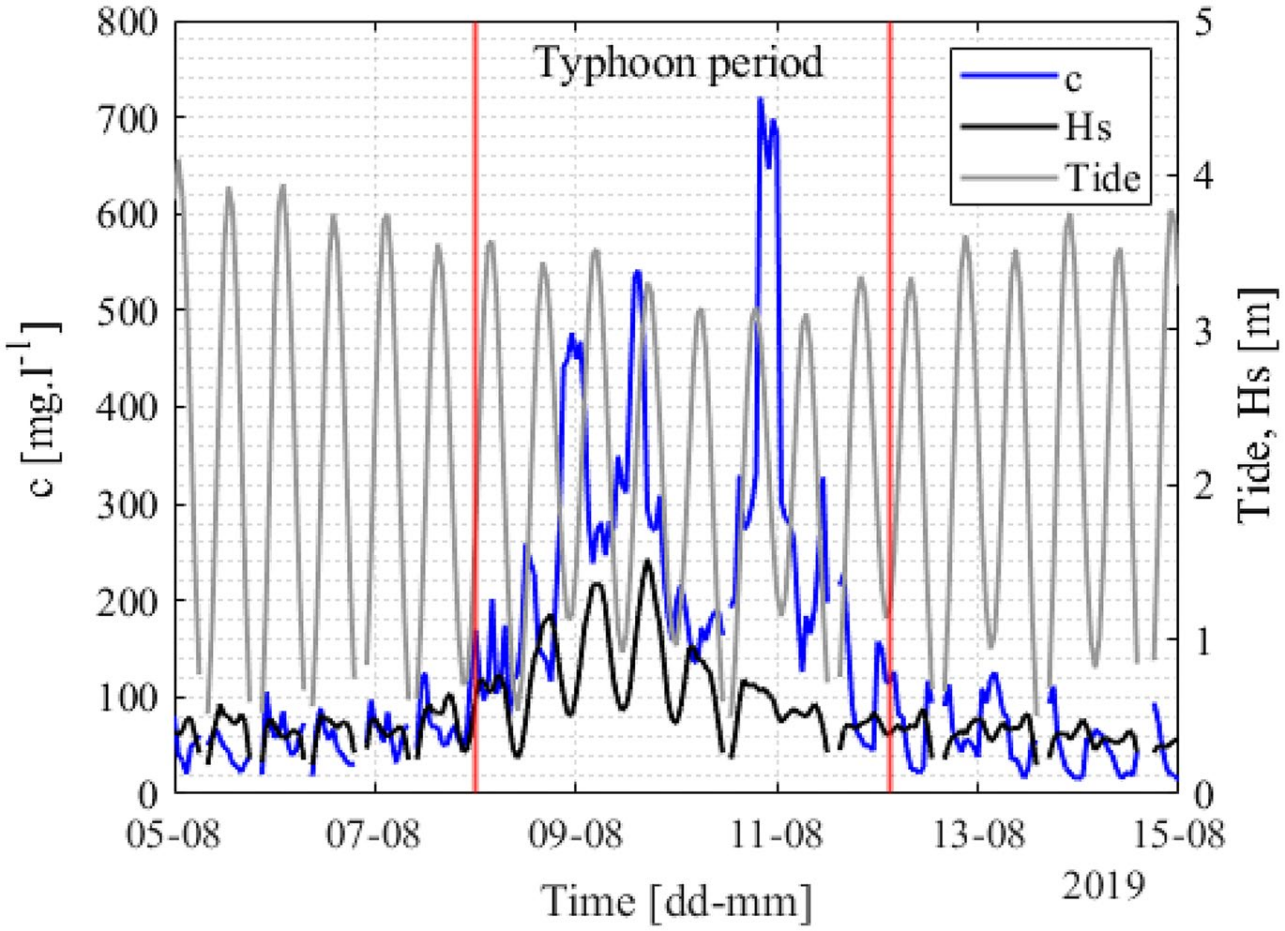
Short periods of *c*, *Hs*, and tide before, during, and after the typhoon at site G2-S. The red vertical lines indicate the typhoon period

The *c* measured from the OBS was consistent with the *c* analyzed from the water sample for the entire monitoring year (Fig. 7). In summer 2019, small waves led to *c* varying from 10 to 90 mg/l at the three monitoring sites (no value at GX-2). During the typhoon period with strong winds and waves in summer, for instance, *c* increased to approximately 500 mg/l. The variation in *c* during the typhoon period is shown in Fig. 8. It showed that *c* rapidly increased under strong wave conditions (*Hs* ~ 1.7 m) compared with typical summer conditions (Fig. 7). The result is reasonable because the rapid increase in *Hs* during the typhoons was the main factor increasing in *c* (Bian et al., 2012). The oscillation in *c* was observed to have a good relationship with waves compared with tides. After the typhoon passed, *c* quickly decreased to the background level (20–80 mg/l). In addition, the monitoring results showed that the average *c* at site G2-L (100.48 mg/l) was higher than that at site GX-1 (74.86 mg/l). The *c* during the winter season was relatively high, ranging from 120 to 300 mg/l. Overall, the results showed that the *c* measured during the winter season was higher than the *c* measured during the summer season. The *c* in this study was significantly higher than the *c* observed in coral reef systems, with a maximum value of approximately 1–8 mg/l (e.g., Pomeroy et al., 2017, 2018).

Table 2 depicts the temporal decomposition of *c* from SSA at the four monitoring sites. The variation in *c* in the two modes represents different driving parameters. At the four monitoring sites, mode 1 from SSA explained approximately 76.41%, 78.51%, 81.26%, and 77.54% of the variance in *c* at the four sites G2-S, G2-L, GX-1, and GX-2, respectively. Mode 2 accounted for 7%, 7.69%, 7.93%, and 5.27% of the *c* variance from the SSA temporal decomposition.

**Fig. 9:**
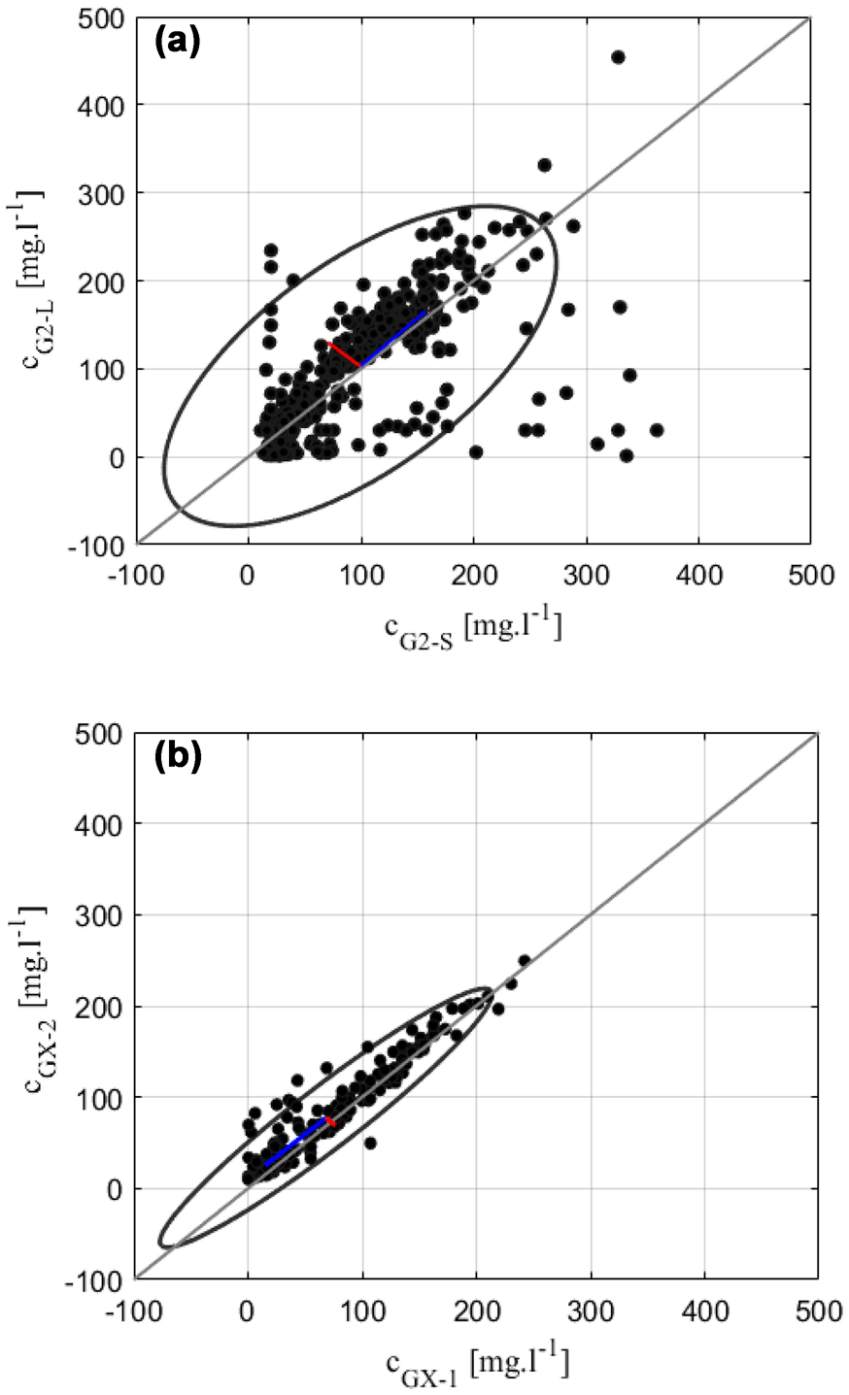
The correlation of daily averaged *c* at the four monitoring sites. Scatter plot of daily averaged *c* at sites **a** G2-S and G2-L and at sites **b** GX-1 and GX-2. The gray line indicates a 1:1 agreement. The black ellipses represent the 95% confidence intervals. The lines shown by red and blue are the eigenvectors of the covariance matrix of the data, whereas the length of the line corresponds to the eigenvalues

The comparison of *c* between the two monitoring Groups G2 and GX is presented in Fig. 9. There was a good comparison between the two monitoring sites G2-S and G2-L (Fig. 9a). However, a weak correlation of *c* was found during some periods between the two monitoring sites due to *Hs* changing at onshore (G2-L) and offshore (G2-S) sites. The *c* measured at site G2-S was higher than that measured at site G2-L, which was possibly caused by differences in the local hydrodynamic forcing at the two monitoring sites (Pomeroy et al., 2018). The variation in *c* between sites G2-S and G2-L is reasonable because it is expected that a smaller *Hs* at site G2-L induces a smaller value of *c* (Brander et al., 2004). Moreover, there was a robust correlation of *c* between sites GX-1 and GX-2 due to the similar wave conditions at these two sites (Fig. 9b). The comparison of eigenvalues (red and blue lines in Fig. 9) between sites G2 and GX showed a significant difference, in which GX had an eigenvalue smaller than that at G2. The results in the correlation of the two nearby sites at GX-1 and GX-2 (Fig. 9b) should be better than the correlation at sites G2-S and G2-L (Fig. 9a). The results also reflect the wave attenuation in the cross-shore direction due to the rough bottom roughness created by the reef (Huang et al., 2012).

## Discussion

### The relationship between wave characteristics and c

**Fig. 10:**
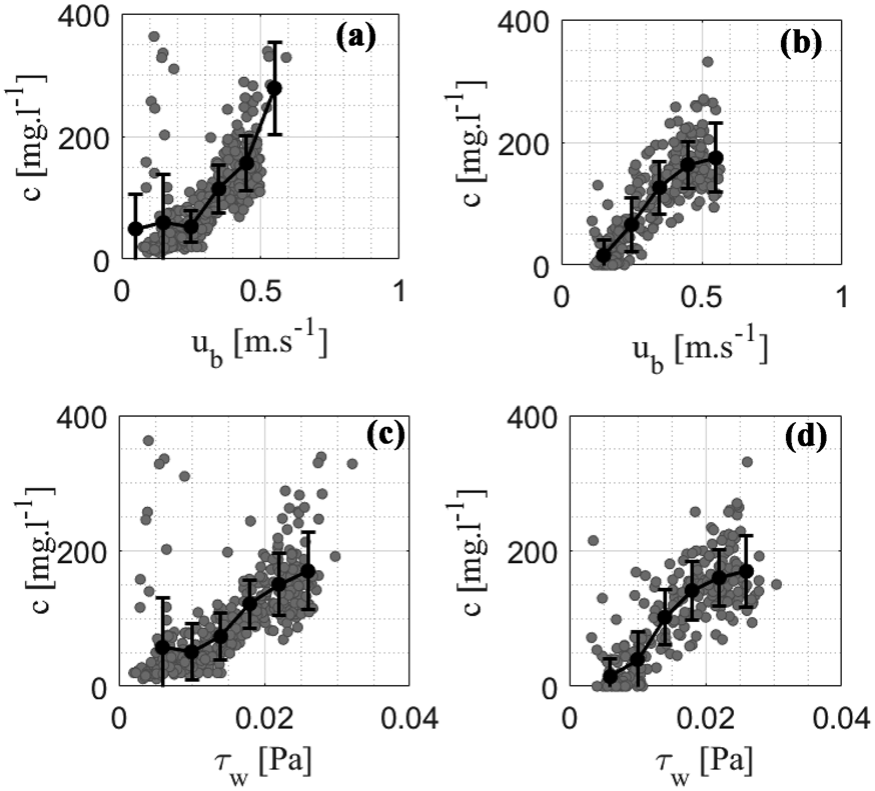
Scatter plots of daily average data of ${u}_{b}$ and *c* at sites **a** G2-S and (b) G2-L, respectively. The two bottom panels show scatter plots of the daily average data of ${\tau }_{w}$ and *c* at sites (c) G2-S and (d) G2-L. The black dots indicate bin averages. The error bars show one standard deviation

As expected, a good correlation was found between ${u}_{b}$ and *c* at both monitoring sites (Fig. 10a and b). As ${u}_{b}$ increased, *c* became denser. However, *c* did not always follow the variability of ${u}_{b}$, which might have been due to several other factors in regard to the hydrodynamic conditions (Pomeroy et al., 2017, 2018). For example, there was a scattered correlation found in the case of ${u}_{b}$ that was small. Clearly, additional work and field experiments are required for a better understanding of the dynamics between waves and sediments. In addition, a weak regression between ${u}_{b}$ and *c* was found when wave breaking occurred (Hashim et al., 2013; Horstman et al., 2014; Huang et al., 2012; Wiberg et al., 2019). The ${u}_{b}$ approaching zero might be caused by wave breaking, resulting in a weak regression between *c* and waves (Wiberg & Sherwood, 2008).

Table 2 illustrates the *c* variation at different time scales via the correlation to ${u}_{b}$ based on the SSA. There was a high correlation between ${u}_{b}$ and *c* (mode 1) at both monitoring sites, G2-S and G2-L, with *R*^*2*^ values of 0.56 and 0.85, respectively. Therefore, the variation in *c* was consistent with the ${u}_{b}$ variability, and there were strong seasonal variations in the two variables. The reason for this phenomenon could be explained by the variation in *c* in the reef system induced by the wave-driven force (Pomeroy et al., 2018). In contrast, mode 2 of ${u}_{b}$ and *c*, caused by the spring-neap tidal regime, showed a weaker correlation. Thus, at this study site, the seasonal variation (mode 1) in wave properties significantly impacted *c* compared with the spring-neap tidal regime (mode 2). This finding agrees well with the conclusion from a previous study in which the role of tides on *c* variation was small (Vo Luong & Massel, 2006).

There was a moderately strong, positive, linear association between the estimated ${\tau }_{w}$ and *c* with a few potential outliers at the two monitoring sites G2-S and G2-L (Fig. 10c and d). A scattered correlation between ${\tau }_{w}$ and *c* was found when ${\tau }_{w}$ was small. Afterward, this correlation became stronger and linear as ${\tau }_{w}$ increased. Table 2 shows the strong relationship between mode 1 of ${\tau }_{w}$ and *c* at two sites, G2-S and G2-L, with *R*^*2*^ values of 0.57 and 0.83, respectively. This robust regression indicated that the variation in both *c* and ${\tau }_{w}$ was a function of the seasons. Therefore, Mode 2 of ${\tau }_{w}$ and *c* had a weaker correlation. Notably, the correlation of *c* and ${\tau }_{w}$ was not always strong because *c* variability depends on multiple factors (Lu et al., 2017; Pang et al., 2020; Pomeroy et al., 2017, 2018), although many correlations obviously exist with the measured near-bed velocity. Even though this study was focused only on wave-induced bed stress, these results can express the contribution of hydrodynamics to the *c* variability following seasonal variation.

## Implications of monitoring waves and c in the algal reef system

Existing studies of sediment dynamics still tend to focus on the relationship between hydrodynamic conditions over the bare sediment bed or large bottom roughness, but only for the coral reef system (Pomeroy et al., 2018). There are very few studies that consider the relation of waves and *c* for long-term monitoring in the algal reef environment. The results from these monitoring systems showed a highly significant inverse relationship between reef health and suspended sediment yields (Jokiel et al., 2014; Miller & Cruise, 1995; Ogston et al., 2004). The threshold of elevated levels of *c* affecting the reef through fertilization, larval survival, and larval settlement is 100 mg/l (Gilmour, 1999). In addition, the tolerance limits of corals in regard to *c* reported in the literature are larger than 100 mg/l in marginal reefs in turbid near-shore environments (Erftemeijer et al., 2012). Therefore, this study assumed that when *c* was continuously higher than 100 mg/l, it could have some negative impacts on reefs in coastal areas. The monitoring of *c* showed the ranges of variation in the following two seasons. The average *c* during the summer season (~ 70 mg/l) was lower than the critical threshold (100 mg/l), while the average *c* during the winter season (~ 140 mg/l) was higher than the critical threshold. Consequently, this may cause considerable damage, such as decreasing reef growth rates and metabolic rates, changing reef population structure and morphology, and reducing species biodiversity and larval settlement (Kuo et al., 2020; Risk & Edinger, 2011).

Hence, it is necessary to develop a combination of feedback monitoring of water quality and reef health during dredging activities to minimize the negative impacts on ambient environmental systems. The most crucial factor mitigating permanent damage is strong water motions, which help remove sediment from algal reefs (Pomeroy et al., 2017; Riegl et al., 1996; Schleyer & Celliers, 2003; Stafford-Smith, 1992). Notably, the hydrodynamics in the study areas will be changed because of the construction of an offshore industrial harbor near the algal reef. It is also challenging to predict increased sediment levels during dredging operations near the LNG station. Some studies have implied that the total *c* transported at a given distance from a dredging operation might increase, depending on the scale of the operation and the conditions of the background water quality (Jones et al., 2016). However, dredging operations that induce elevated *c* values that affect algal reef systems and natural disaster events, such as typhoons, also need to be accounted for (Erftemeijer et al., 2012).

The *c* on coasts with high roughness regions caused by reefs in this study was mostly due to waves (Pomeroy et al., 2018). Here, we first focused on the setup of the long-term monitoring of *c* in coastal areas, even though the interaction between hydrodynamic conditions and *c* variability is still complicated. Therefore, detailed coupling studies on hydrodynamic and sediment transport, such as wave-current interactions and turbulence properties, are needed. Even though the results from this research showed a strong relationship between *c* variation and wave properties, the impact of currents should also be considered. Due to the limitation of in situ measurements, this study recommends that further research be conducted to improve estimations of the bed shear stress induced by both currents and waves to precisely predict *c* variation. In addition, the spatial sediment variability, including the concentration and particle size of the sediment at different elevations in the water column, should be surveyed to develop robust sediment transport equations that can be applied to other intertidal reef ecosystems.

## Conclusions

We have presented a system for the real-time and long-term monitoring of waves and suspended sediment concentrations, *c*, in intertidal algal reefs. The system was based on OBS, pressure sensor, data logger, and wireless network techniques and is continuously and automatically operated. The monitoring project has been successfully executed for more than three years and is aimed for a decade during the construction and operation periods of an LNG receiving port. The long-term data are useful for assessing the environmental impacts of possible changes in hydrodynamics and sediment transport.

Assuming the data of *c* measured by water sampling are ground-truth values, the OBS measurement was compared and validated with biweekly water sampling. A good correlation was observed between the results of the two methods. From the year-long data, we tested the number of calibrations of the OBS from the four seasons. Nevertheless, more calibrations of the OBS in different seasons reduced the variance between the two methods over a year-long timescale.

The year-long data remarkably showed the existence of seasonal variations in *c* and wave properties over the algal reef. The results from singular spectrum analyses showed that the mode of the seasonal component prevailed over the spring-neap tide component in the year-long time-series data of *c* and wave properties. High values of *c* were found during the winter season under high wave conditions. The average *c* was approximately 70 mg/l during the summer season and approximately 140 mg/l during the winter season. The observed values of *c* ranged from approximately 0 to 500 mg/l, which were much higher than those in other coral reef environments. The elevated and highly variable *c* may be one factor that makes the unique algae reef environment (Yu et al., 2020). We also found that *c* was highly correlated with the wave orbital velocity, ${u}_{b}$, and the wave-induced bed shear stress, ${\tau }_{w}$. The presented system and the long-term observed data will definitely help in assessing the environmental change in the reef habitat and will provide useful data for mitigating, improving, and restoring the algal reef system.

## Availability of data

Processed data supporting the figures can be found from the corresponding author on reasonable request (zchuang@ncu.edu.tw).

## Appendix

Acronyms

**Table:**

G2-S	Seaward monitor system, site G2
G2-L	Landward monitor system, site G2
GX-1	Monitor system 1, site GX
GX-2	Monitor system 2, site GX
*Hs*	Significant wave height
CCA	Crustose coralline algae
*RMS*	Root mean square
*RMSE*	Root mean square error
${u}_{b}$	RMS near-bottom orbital velocity
*c*	Suspended sediment concentration
OBS	Optical backscatter sensor
MOXA	Real-time internet transmission system
ADP	Acoustic Doppler profiler
LNG	Liquefied natural gas
${k}_{w}$	Nikuradse roughness
${\sigma }_{b}$	Bottom roughness
${\omega }_{r}$	Radian frequency
${\tau }_{c}$	Current shear stress
${\tau }_{w}$	Wave bed stress
${f}_{w,r}$	Wave friction factor
${a}_{n}$	Amplitude of the *n*^th^ component
*ρ*	Water density
${u}_{b,n}$	Near bottom orbital velocity of the *n*^th^ component
